# Attracting Nurses to Practice in Skilled Nursing Home Settings With Age-Friendly Curriculum

**DOI:** 10.1093/geroni/igaf122.2185

**Published:** 2025-12-31

**Authors:** Diane Brown, Sue Hazelett, Susan Fosnight, Jennifer Drost, Cynthia Hovland, Margaret Sanders, Lori Kidd, Donna Barrett

**Affiliations:** The University of Akron, Akron, Ohio, United States; Summa Health System, Akron, Ohio, United States; Summa Health System, Akron, Ohio, United States; Summa Health System, Akron, Ohio, United States; Cleveland State University, Cleveland, Ohio, United States; Northeast Ohio Medical University, Rootstown, Ohio, United States; The University of Akron, Akron, Ohio, United States; Summit County Public Health, Akron, Ohio, United States

## Abstract

Skilled nursing home quality of care and staffing are positively correlated. Staffing shortages have been a long-standing problem with more than one in four US nursing homes reporting nurse shortages. We developed a 1-credit course for undergraduate nurse seniors with 12 online modules based on Age-Friendly Health Systems care, interprofessional teamwork, and other skilled nursing setting relevant topics. Modules included a short video lecture, pre-post quizzes, discussion boards, and supplemental materials. Trainees also spent 10 hours shadowing the interprofessional team in a skilled nursing facility. Forty students completed the course and pre/post course surveys to measure outcomes. Statistical path analysis was done using survey items regarding practice environment factors and interprofessional teamwork attitudes to assess for predictors of increasing a student’s willingness to work with older adults, or to work in the skilled nursing setting. Results identified if giving psychological support was rated with high importance, the student had an 85% chance to increase their interest in the care of older adults post-course. If a student rated making clinical decisions with high importance, they were 88% more likely to increase their interest in practicing in a nursing home setting post-course. Qualitatively, students cited the negatives that deter interest in nursing home work most commonly as nurse-patient ratios, low pay, emotional toll of frequent deaths, and working with dementia clients. These results can help identify ways to increase recruitment of nurses to work with older adults and in skilled nursing facilities.

